# Oscillations and chaos in the dynamics of the BCM learning rule

**DOI:** 10.1186/1471-2202-14-S1-P285

**Published:** 2013-07-08

**Authors:** Lawrence C Udeigwe, G Bard Ermentrout, Paul W Munro

**Affiliations:** 1 School of Information Sciences, University of Pittsburgh, Pittsburgh, PA 15260, USA; 2 Department of Mathematics, University of Pittsburgh, Pittsburgh, PA 15260, USA

## 

The BCM learning rule originally arose from experiments intended for measuring the selectivity of neurons in the primary visual cortex, and it dependence on input stimuli. This learning rule incorporates a dynamic LTP threshold, which depends on the time averaged postsynaptic activity. Although the BCM learning rule has been well studied and some experimental evidence of neuronal adherence has been found in the other areas of the brain, including the hippocampus, there is still much to be known about the dynamic behavior of this learning rule.

The dynamics of BCM cell can be described as follows:

$$\tau_{w}\frac{dw}{dt}=\nu x_{j}^{\left(i\right)}\left(\nu -\theta \right)$$

$$\tau_{\theta }\frac{d\theta }{dt}=\nu^{2}-\theta$$

where $x^{\left(i\right)}=\left(x_{1}^{\left(i\right)},...,x_{n}^{\left(i\right)}\right)$ is an input stimulus pattern, and $w=\left(w_{1},...,w_{n}\right)$ is the synaptic weights. The postsynaptic activity is computed as $\nu =w.x^{\left(i\right)}$, and  θ is a "sliding" threshold for the postsynaptic activity. and are constants.

**Figure 1:**
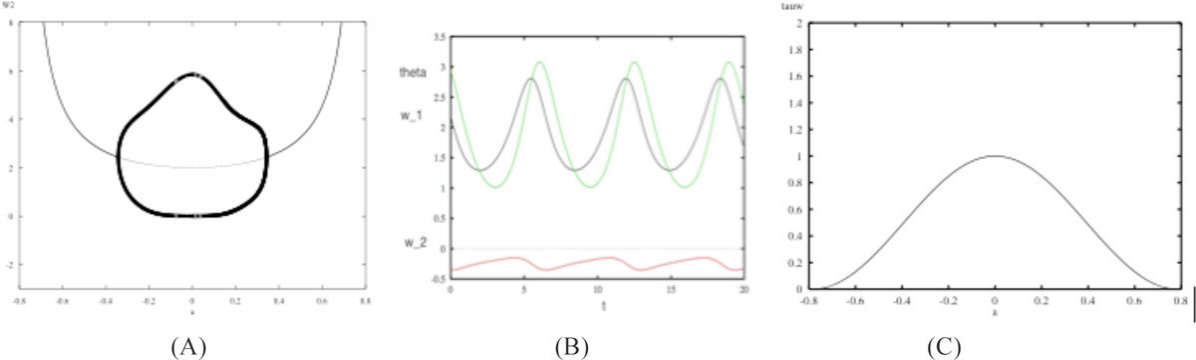
(A) w_1_-vs-a, where a is the parameterizes of input stimulus. The bold curve shows the periodic orbits as a varies. (B) The green curve is θ-vs-time, the black curve is w_1_-vs-time, and the red curve is w_2_-vs-tme. Each exhibiting a stable oscillation when α = 0.128 and τ_w_ (C) Two parameter curve of Hopf bifurcations, τ_w_-vs-a. The weights exhibit a winner-take-all behavior in the region above the curve, and an oscillatory behavior in the region below the curve. In each subfigure, *τ_θ_*.

In this work, a mean-field version of the BCM learning rule is studied, and it is shown that if the synaptic weights and the postsynaptic activity threshold share similar time scales, then it is possible to obtain complex dynamics. It is also shown that there exist periodic orbits for certain parametric regions of stimulus orientation and time-scale factor, as evidenced by a Hopf Bifurcation (see Figure 1). Consequently, it is discovered that the synaptic weights exhibit an oscillatory behavior in this region. A preliminary study of two BCM cells coupled by lateral inhibition yields a torus bifurcation, which tends to lead to chaos.

